# The Lack of Standardization and Pharmacological Effect Limits the Potential Clinical Usefulness of Phytosterols in Benign Prostatic Hyperplasia

**DOI:** 10.3390/plants12081722

**Published:** 2023-04-20

**Authors:** Mădălina-Georgiana Buț, George Jîtcă, Silvia Imre, Camil Eugen Vari, Bianca Eugenia Ősz, Carmen-Maria Jîtcă, Amelia Tero-Vescan

**Affiliations:** 1 Doctoral School of Medicine and Pharmacy, I.O.S.U.D, George Emil Palade University of Medicine, Pharmacy, Science and Technology of Târgu Mures, 540139 Târgu Mures, Romania; madalina-georgiana.batrinu@umfst.ro (M.-G.B.); carmenrusz20@gmail.com (C.-M.J.); 2 Department of Biochemistry, Faculty of Pharmacy, George Emil Palade University of Medicine, Pharmacy, Science and Technology of Târgu Mures, 540139 Târgu Mures, Romania; amelia.tero-vescan@umfst.ro; 3 Department of Pharmacology and Clinical Pharmacy, Faculty of Pharmacy, George Emil Palade University of Medicine, Pharmacy, Science and Technology of Târgu Mures, 540139 Târgu Mures, Romania; camil.vari@umfst.ro (C.E.V.); bianca.osz@umfst.ro (B.E.Ő.); 4 Department of Analytical Chemistry and Drug Analysis, Faculty of Pharmacy, George Emil Palade University of Medicine, Pharmacy, Science and Technology of Târgu Mures, 540139 Târgu Mures, Romania; silvia.imre@umfst.ro

**Keywords:** phytosterol, benign prostatic hyperplasia, sitosterol, campesterol, dietary supplement

## Abstract

The prevalence of benign prostatic hyperplasia (BPH) markedly increases with age. Phytotherapeutic approaches have been developed over time owing to the adverse side effects of conventional medications such as 5-reductase inhibitors and α1-adrenergic receptor antagonists. Therefore, dietary supplements (DS) containing active compounds that benefit BPH are widely available. Phytosterols (PSs) are well recognized for their role in maintaining blood cholesterol levels; however, their potential in BPH treatment remains unexplored. This review aims to provide a general overview of the available data regarding the clinical evidence and a good understanding of the detailed pharmacological roles of PSs-induced activities at a molecular level in BPH. Furthermore, we will explore the authenticity of PSs content in DS used by patients with BPH compared to the current legislation and appropriate analytical methods for tracking DS containing PSs. The results showed that PSs might be a useful pharmacological treatment option for men with mild to moderate BPH, but the lack of standardized extracts linked with the regulation of DS containing PSs and experimental evidence to elucidate the mechanisms of action limit the use of PSs in BPH. Moreover, the results suggest multiple research directions in this field.

## 1. Introduction

Benign prostatic hyperplasia (BPH) is a common and progressive condition that affects the quality of life of men in an age-dependent manner, being present in 15–60% of men over 40 years [1,2,3]. BPH is a non-malignant enlargement of the prostate that can lead to obstruction and irritation of the lower urinary tract. As the prostate gland increases in volume, constriction of the urethra occurs with the appearance of symptoms such as weak urinary flow, incomplete emptying of the bladder, nocturia, or dysuria. These symptoms are associated with BPH and are referred to as lower urinary tract symptoms (LUTS) [4]. The etiology of BPH is influenced by a wide variety of risk factors, such as age, hormonal imbalance, inflammation, metabolic syndrome, oxidative stress, or inhibition of apoptosis in prostate tissue [4].

Reducing LUTS and improving the quality of life (QL) are the primary goals of BPH treatment. As therapy has changed significantly over the last decade, the number of surgeries has steadily decreased while the number of cases treated with medicine has increased [5,6]. Currently, two large classes of drugs are used in pharmaceutical practice for the treatment of BPH: α1-adrenergic receptor antagonists (doxazosin, terazosin, and tamsulosin), which relieve LUTS by relaxing the smooth muscle of the prostate stroma and bladder neck, and 5α-reductase inhibitors (5-αRi) such as finasteride or dutasteride [7,8]. 5α-reductase (5-αR) is an enzyme of major importance in the development of BPH and is responsible for the formation of dihydrotestosterone (DHT), the main biologically active metabolite of testosterone (T). It is well known that androgen excess, mainly DHT, has been suggested to be associated with the development of BPH [9,10]. However, these prescription-only medicines are used in advanced forms of BPH, according to the attending physician’s recommendation. Although there is a significant clinical benefit when administered to BPH patients [11,12], conventional therapy is correlated with disorders of sexual dynamics in men, such as erectile dysfunction, increased risk of impotence, ejaculation disorders, gynecomastia, and orthostatic hypotension [11,13,14,15].

Growing interest in the use of dietary supplements (DS) for healthcare management has led to the availability of natural products in the market. Consequently, an increasing number of patients tend to adopt a plant-based treatment for BPH. Recent data present a wide range of bio-compounds, such as phytosterols, phenolic compounds (polyphenols, catechins), and fatty acids, which can be further formulated as adjuvant medication to conventional therapy. For instance, phytosterols (sitosterol, stigmasterol, campesterol), which have a similar chemical structure to synthetic 5-αRi, represent the most promising class derived from plants with inhibitory action on 5-αR. Phytosterols (PSs) are increasingly used to alleviate BPH symptoms, and DS manufacturers promote their beneficial effects without substantial experimental evidence. The National Institutes of Health (NIH) Dietary Supplement Label Database (DSLD) releases a database with info on over 5000 dietary supplement labels for supporting healthy prostate function in men [16]. The most common sources of PSs found in DS are *Serenoa repens* (54%), *Pygeum africanum* (15%), *Urtica dioica* (14%), *Curcupita pepo* (14%), or sitosterol in the singular form [17]. Available published data focus mainly on establishing and demonstrating the beneficial effects of plant-based DS in BPH [3,18,19]; however, the underlying mechanisms of PSs involved have not been thoroughly investigated.

As over-the-counter medications, PS-based DS are available in pharmacies, natural stores, or online and information accessible to users is skewed toward commercial interests, making them vulnerable to hyperbolic advertising and misleading claims. Since the regulatory aspects of the DS industry are ambiguous and incomplete, manufacturers set their own standards. Our understanding of plant extracts is limited because of the lack of standardization. Thus, clinical trials are difficult to interpret, and the therapeutic efficacy of phytocompounds is inconsistent, resulting in a lack of progress in clinical practice and public health [20]. In this context, another difficulty is brought about by challenges in the analytical field, and the lack of standardized analysis techniques for monitoring the quality of PSs in DS is absolutely necessary.

In light of the growing BPH burden around the world, the aim of this study is to investigate the role of PSs in BPH. We sought to provide the limitations and difficulties associated with using PSs for BPH, considering the potential therapeutic benefits of PSs, their low pharmaco-toxicological profile, the difficulty in assessing their clinical effectiveness, as well as the growing interest in the consumption of PSs. Thus, the purpose of this paper is to review the molecular-level mechanisms of action of PSs intervening in BPH and the clinical evidence, the quality and quantity of PSs used by patients with BPH compared to the legislation in force, and the appropriate analytical methods reported in the literature used for qualitative and quantitative monitoring of DS with PSs content.

To the best of our knowledge, this is the first study to assess, in particular, the effects of phytosterols from multiple views in BPH. To fill a gap in the existing literature and uncover the nature of review novelty, critical building blocks include chemical properties, sources of PSs, analytical methods for tracking DS containing PSs, regulatory framework, molecular mechanisms, and clinical evidence for PSs in BPH.

## 2. Phytosterols—Characteristics

### 2.1. Chemical Properties of Plant Sterols

**Figure 1:**
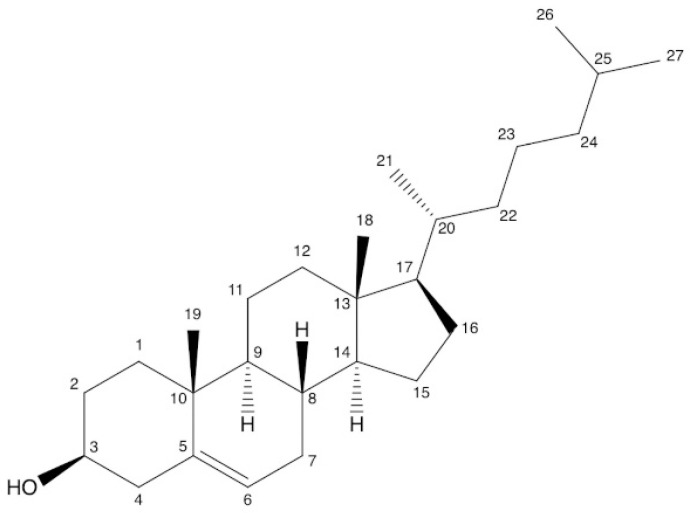
Cholesterol with carbon numbering according to IUPAC.

**Figure 2:**
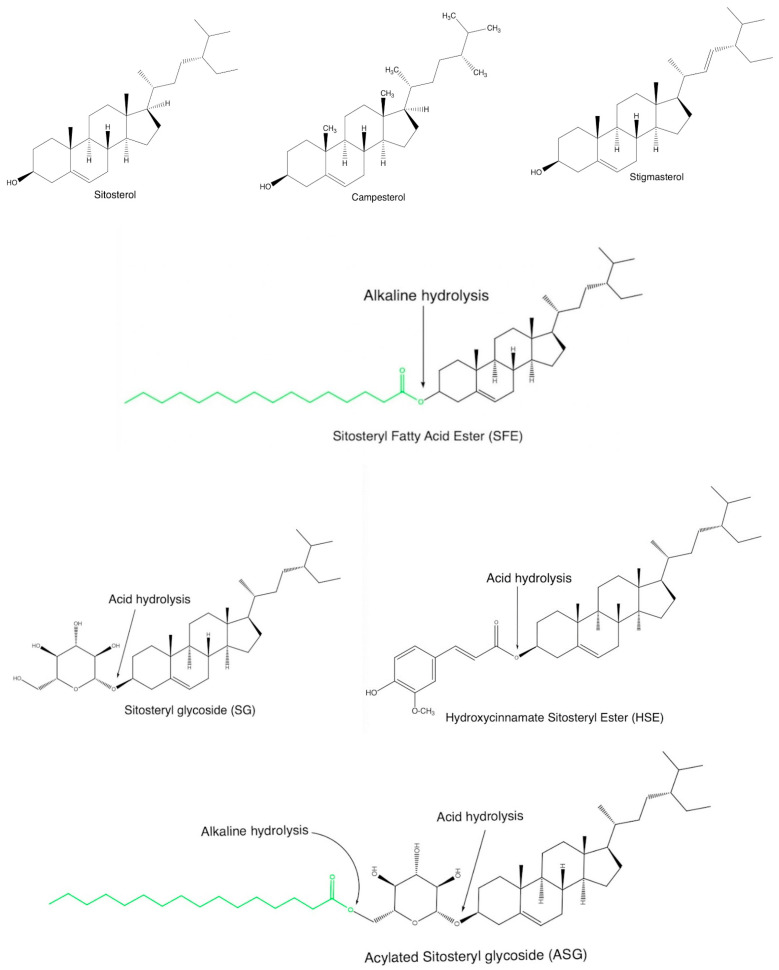
The structures of the most common phytosterols found in dietary supplements (free-form FPS and conjugated CPS); green radical—fatty acid residue.

Structurally, PSs are bioactive compounds and plant equivalents of cholesterol that are specific to animal groups. Steroidal in shape, they contain a hydroxyl group at C_3_, a hydrocarbonate chain at C_17_, and one or more double bonds at C_5_ of the base skeleton [21,22,23], see Figure 1 and Figure 2. Phytostanols are a subgroup of this class, with a fully saturated basic skeleton [23,24].

Despite the presence of over 200 PSs, β-sitosterol (SIT), stigmasterol, and campesterol, are the essential phytosterols to consider. As the dominant compound, SIT accounted for 90% [23]. These data were confirmed by NIH-DSLD, which released label statements with DS containing mostly SIT, campesterol, and stigmasterol. However, most previous studies have focused on the same phytosterol for various effects, such as anti-obesity, anti-diabetic, anti-microbial, anti-inflammatory, immunomodulatory, and anti-cancer effects [16].

PSs can be found in a free form (FPS) or conjugated form (CPS) at the hydroxyl group from position 3 (Figure 1). The four common types of conjugate sterol lipid classes are sitosteryl fatty acid ester (SFE) which is the most common, sitosteryl glycoside (SG), acylated, sitosteryl glycoside (ASG), or hydroxycinnamate sitosteryl ester (HSE) [25,26] (Figure 2). There is no evidence that CPS has the same therapeutic effects as FPS in BPH, with CPS being less studied due to the lack of analytical reference standards. Therefore, PSs analysis should consider the form of the PSs present in the matrix (FPS or CPS) and use a suitable extraction method to ensure the determination of the total amount of PSs. Instead, CPS has a lower melting point and higher solubility in vegetable oils [27] and is used to enrich foods, such as salad dressings [28], margarine [29], or cheddar [30].

### 2.2. Sources of Plant Sterols

PSs are ubiquitous compounds distributed in nature and are present in small amounts in daily food. As dietary sources, fruits and vegetables usually contain only small amounts of PSs (less than 0.05% on a wet basis) [25,26]; however, nuts and vegetable oils may contain more than 1% PSs [31]. On the other hand, phytostanols are present in cereals (corn, wheat, rye, rice), fruits, and vegetables, but their concentrations are generally lower than unsaturated plant sterols [32]. The typical daily consumption of PSs is between 140–400 mg/day in different populations depending on the country and type of diet, and it comes mostly from vegetable oils, cereals, fruits, and vegetables [33]. Average PSs ratios between 140–360 mg/day have been estimated in Finland; for a review, see [23,34], 163 mg/day United Kingdom [23], 100.6 mg/zi Brazil [35], or 392.3 mg/day in China [36]. Vegetarians, generally vegans, have the highest PSs intake of >1 g/day [37]. In DS, the main source of PSs is represented by standardized plant extracts, either as a single compound or as a combination of active principles. Dwarf palm extract (*Serenoa repens*) is the most commonly used DS formulation [38]. Consequently, the National Institute of Standards and Technology (NIST) has developed two standard reference materials (SRM), SRM 3250 *Serenoa fruits* and SRM 3251 *Serenoa repens*, which can be used as a reference for PSs monitoring in DS with *Serenoa repens* extract [39]. In addition to dwarf palm extract, other plant sources containing PSs used in BPH are *Curcubita pepo* seeds, *Epilobium parviflorum*, *Epilobium angustifolium* aerial parts, *Hypoxis hemerocallidea*, *Hypoxis rooperi* corn, *Prunus africana* bark, *Secale cereale* pollen, *Urtica dioica* root, and *Cernilton* [3,19].

## 3. Phytosterols—Beneficial Effects in BPH

### 3.1. Pathophysiology of Benign Prostatic Hyperplasia

Solid and complex pathophysiological backgrounds are involved in BPH. BPH is characterized by the proliferation of both stromal and epithelial elements, leading to gland enlargement and, in some cases, urinary obstruction [40]. Although the cause of BPH remains incompletely elucidated, it is known that a central role is played by the growth of both stromal and epithelial elements caused by excess androgens [41]. BPH does not occur in males castrated before puberty or in those with genetic diseases that block the activity of androgens [42]. The action of DHT is mediated via androgen receptors (ARs), a ligand-dependent nuclear transcription factor and a member of the steroid hormone nuclear receptor family, which regulates the expression of genes that support the growth and survival of prostatic epithelial and stromal cells. Although T can also bind to ARs and stimulate growth, DHT is ten times more potent [43].

In addition, the male body stimulates the conversion of T to estrogen (ES) by increasing aromatase activity due to the high percentage of adipose tissue associated with advancing age. The circulating ES/T ratio increases, resulting in a decreased negative androgen control of ES release [4,44]. Estrogen also plays an important role in the development of BPH, and its level is correlated with prostate volume [45]. In contrast, the study conducted by Miwa et al. did not identify the same relationship between ES levels and prostate volume [46]. Estrogen receptor (ER) types are thought to have a major influence on the effects of ES on prostate tissues. Specifically, ER-α stimulation causes hyperplasia, dysplasia, and inflammation [47]. Conversely, ER-β activation decreased proliferation and promoted apoptosis in BPH in an androgen-independent manner [48]. Therefore, ER-α/ER-β ratio plays an important role in ES-induced cell proliferation.

Several previous experimental studies have linked chronic inflammation with the development of BPH, suggesting that chronic inflammation may contribute to the development of the disease [49,50,51]. The role of inflammation in the development of BPH is underlined by the strong correlation between inflammation proven by histological criteria, the International Prostate Symptom Score (IPSS), and prostate volume; thus, the inflammatory process is considered a therapeutic target in BPH [49]. A study conducted by Nickel et al. analyzed prostate tissue from 374 patients who underwent transurethral resection of the prostate (TURP) for BPH and noted the presence of chronic or acute inflammation in 70% of patients [52]. Cyclooxygenase-2 (COX-2) expression is associated with inflammatory processes in BPH. Prostaglandins are a group of pro-inflammatory mediators, synthesized from arachidonic acid, under the action of COX-1 and COX-2, identified in the prostate tissue of BPH patients [53,54]. Furthermore, a study by Wang and his colleagues suggested that up-regulation of anti-apoptotic proteins correlated with increased COX-2 expression inhibits prostate apoptosis in BPH [55]. Other studies have demonstrated that the administration of COX-2 inhibitors to patients with BPH produces a significant increase in apoptotic processes [56]. Additionally, another study has demonstrated that induced nitric oxide synthase (iNOS) is only present in patients with BPH and contributes to inflammation [57].

Oxidative stress is another trigger that can lead to BPH development and progression. A study using an animal model of BPH reported decreased activity of antioxidant systems such as glutathione, superoxide dismutase, glutathione peroxidase, and catalase. Furthermore, a significant increase in the lipid peroxidation process in BPH has been reported, which was inhibited by finasteride administration [58]. Considering the pathophysiological implications, the use of PSs in BPH is explained by their anti-androgenic, anti-inflammatory, and antioxidant effects alongside their ability to modulate apoptotic processes.

### 3.2. Molecular Mechanism of PSs in BPH Development

- Anti-androgenic effect of phytosterols in BPH

Plant-derived PSs that are structurally similar to synthetic 5-αRi (finasteride, dutasteride) represent a potentially highly effective class of 5-αRi. According to an in vitro study performed by Marisa Cabesa and her colleagues on hamster prostate tissue, SIT inhibits 5-αR in a dose-dependent manner (IC_50_ = 1.1 μg/mL compared to finasteride with IC_50_ = 0.003 μg/mL) [59]. Other data have shown that stigmasterol, extracted from *Phyllanthus urinaria*, also inhibits 5-αR and is less potent than SIT (IC_50_ = 11.2 μg/mL) [60]. To offset the low efficacy of PSs, a combination of conventional treatments with PSs may provide some clinical benefit. Studies have also been performed on plant extracts containing PSs with inhibitory action on 5-αR, without the individual identification and specific contribution of each phytocompound to 5-αR activity [61,62,63]. The results are summarized in Table 1. Among the extracts containing PSs, saw palmetto extract has been the most studied. An in vitro study performed on *Serenoa repens* extract using a baculovirus-directed insect cell expression system demonstrated its ability to inhibit 5-αR in a non-competitive manner [64]. Various saw palmetto extracts (hexane, ethanol, and hypercritical CO_2_) have reported IC_50_ values between 25 µg/mL and 2200 µg/mL, depending on the modality of 5-αR activity assessment [65]. The data collected indicate that SIT, a standardized extract, is more potent than non-standardized extracts from plants, despite having a potency over 1000× lower than finasteride. Moreover, the extraction method used may affect the inhibitory activity of phytocompounds from plant products. A study conducted by Nahata et al. analyzed the inhibitory capacity of 5-αR on different extracts from *Urtica dioica* root (ethanolic, petroleum ether, and aqueous); the ethanolic extract was the most potent [66].

In addition to the 5-αR inhibitory activity, PSs resulted in decreased AR expression and inhibition of the DHT-AR complex [67]. The estrogenic effects of PSs have recently been reviewed by Nattagh-Eshtvani E. Furthermore, colleagues [68]. According to an in vivo study, PSs have direct estrogenic activity and act as selective modulators of ES (SERMs) on both ER-α and ER-β. Although PSs interact with the ER, the administration of SIT in an animal model did not increase uterine weight, a key marker of estrogenic activity; for a review, see [68,69,70]. However, in BPH, there are no data proving the beneficial or non-beneficial effects of PSs through an estrogen-mediated mechanism; the estrogenic effect at the level of prostate tissue depends on the ability of PSs to stimulate ER but also on the ER-α/ER-β ratio.

- Phytosterols as anti-inflammatory and antioxidant dietary components in BPH

The anti-inflammatory effects of PSs have been studied and demonstrated in animals and humans under various pathological conditions. In a study performed in rats with non-alcoholic fatty liver disease, plant sterol-fortified skimmed milk was administered. After 12 weeks of treatment, inhibition of interleukin 6 (IL-6), interleukin 10 (IL-10), and C-reactive protein and improvement of hepatic steatosis were observed [71]. Researchers have found that treatment with SIT (20 mg/kg) for 8 weeks inhibited the activation of nuclear factor kappa B (NF-kB) and the production of pro-inflammatory cytokines in mice with high-fat-diet-induced intestinal injury and inflammation [72].

It is well known that NF-kB stimulates the expression of proteins that contribute to the pathogenesis of inflammation. NF-kB activation is a hallmark of inflammation [73]. Inhibition of NF-kB activation by PSs has been reported by several authors [74,75,76]. Moreover, recent data indicate that PSs alleviate the inflammatory reaction in lipopolysaccharides (LPS)-induced macrophage models and cell phagocytosis and inhibit the release of tumor necrosis factor-α (TNF-α), the expression and activity of COX-2, iNOS, and phosphorylated extracellular signal-regulated protein kinase (p-ERK). The anti-inflammatory activity of SIT was higher compared to that of stigmasterol and campesterol, suggesting that PSs without a double bond at C_22_ and with an ethyl group at C_24_ are more potent anti-inflammatory agents [77].

Based on the relationship between inflammation and BPH, an in vivo study tested the anti-inflammatory properties of *Serenoa repens* extracts enriched with 3% SIT (0.2–0.3% normally). The results showed that SIT significantly decreased the expression of COX-2 compared to the untreated BPH group [78]. In the same study, the group with BPH without treatment showed increased levels of NF-kB compared to the placebo group, and in the case of the group treated with *Serenoa repens* extract enriched with SIT, the gene expression of NF-kB was significantly inhibited [78]. Therefore, the anti-inflammatory effect of SIT in BPH is due to the inhibition of COX-2 and NF-κB expression. No studies have strictly evaluated the anti-inflammatory effects of PSs in BPH subjects.

Its anti-inflammatory effect is closely related to its antioxidant effects. Inflammation is one factor that produces reactive oxygen species (ROS) in the prostate tissue and is associated with oxidative stress [79], and an excess of ROS can also trigger the inflammatory process [80,81]. PSs have an antioxidant effect and act as free radical scavengers, cell membrane stabilizers, and antioxidant enzyme boosters; for a review, see [82,83]. However, in BPH, the antioxidant effects of PSs have not yet been tested. At the hepatic level, SIT attenuates alcohol-induced ROS by restoring erythrocyte membrane fluidity, reducing glutathione depletion malondialdehyde overproduction, restoring antioxidant enzyme activity, and reducing malondialdehyde overproduction [84].

- Apoptotic effect of phytosterols in BPH

Cell growth is controlled by a constant balance between the stimulation and inhibition of apoptosis-related metabolic pathways. In particular, the Bcl-2 family of proteins (B-cell lymphoma 2) and the Bcl-2 associated with protein X (BaX) play an essential role by modulating the activity of certain caspases, especially caspase-9. Bax-expressing cells undergo apoptosis, while Bcl-2-expressing cells undergo carcinogenesis, which results from suppressing apoptosis [85]. Specifically, increased levels of Bcl-2 inhibit Bax and prevent cytochrome C from being released into mitochondria, which inhibits the formation of necessary complexes of apoptotic protease activating factor-1 (APAF1), cytochrome C, and caspase-9, essential for cell survivor [86]. The Bax/Bcl-2 ratio has been suggested to play an important role in the regulation of apoptosis by androgens. Thus, androgen deficiency during apoptosis led to an increase in the Bax/Bcl-2 ratio [87]. Several studies have suggested that apoptosis is diminished in BPH. For example, a study conducted by Kyprianol et al. showed increased expression of Bcl-2 in epithelial cells compared to that in healthy prostate tissue [88]. Furthermore, other studies have shown increased levels of apoptotic inhibitory proteins in the human prostate with BPH [89,90].

Another pathway involved in the regulation of apoptosis is mitogen-activated protein kinase (MAPK). Three MAPK pathways are involved in cell cycle modulation: extracellular signal-regulated protein kinase (ERK), p38, and Jun N-terminal Kinase (JNK) [91]. JNK and p38 synergistically promote apoptosis [92]. JNK-dependent apoptosis is inhibited by ERK-MAPK activation [93]. JNK can induce Bax phosphorylation, promote mitochondrial translocation, promote apoptosis, and inactivate Bcl-2 [94]. In BPH, there is an increased activity of the ERK cascade and a suppressive effect on the JNK and p38 pathways [67,95]. This is where the hormonal imbalance involved in BPH comes into play. Overexpression of EGF and IGF proteins has been observed in BPH [96].

It is assumed that excess DHT leads to the production of growth factors, particularly epidermal growth factor (EGF) and insulin growth factor (IGF) [97]. In prostate tissue, EFG and IGF receptor activation cause ERK/MAPK activation and consequently inhibit JNK-promoted apoptosis [98]. Transforming growth factor beta (TGF-β) is another growth factor that plays a beneficial role in BPH by inhibiting the proliferation process and promoting apoptosis in epithelial cells [99]. TGF-β activity can be influenced by DHT [67,97].

**Figure 3:**
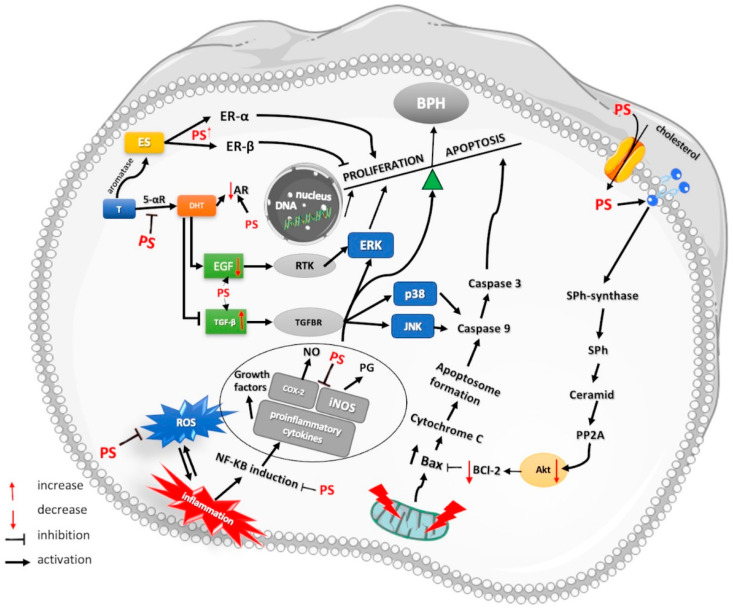
Schematic representation of mechanism involved in BPH. Anti-androgenic, anti-inflammatory, antioxidant, and pro-apoptotic mechanisms of PSs. PS: phytosterols, ES: estrogen, T: testosterone, DHT: dihydrotestosterone, Bcl-2: B-cell lymphoma 2, Bax: Bcl-2- associated X protein, ERK: extracellular signal-regulated protein kinase, JNK: Jun N-terminal Kinase, EGF: epidermal growth factor, TGF-β: transforming growth factor β, PP2A: phosphatase A, 5-αR: 5α-reductase, COX-2: cyclooxygenase-2, iNOS: inducible nitric oxide synthase, RTK: receptor tyrosine kinase, TGFBR: TGF-β receptor, ROS: reactive oxygen species; PG: prostaglandins; Akt: protein kinase B; SPh: sphingomyelin; Sph-synthase: sphingomyelin synthase.

Based on the previews available to date, an animal model with BPH demonstrated the anti-BPH activity of PSs extracted from *Cucurbita pepo* by regulating the balance between proliferative and apoptotic processes. The study demonstrated that administration of PSs increased TGF-β expression and prevented ERK activation, thus promoting apoptosis through caspase 3 activation due to JNK and p38 phosphorylation [67]. According to another study, *Serenoa repens* extract enriched with 3% SIT possesses pro-apoptotic action in the prostate tissue of rats by inhibiting pAkt [78]. Akt, also known as protein kinase B, phosphorylates threonine and serine residues in target proteins. Akt activation increases the expression of anti-apoptotic stimuli (Bcl-2) through cAMP response element-binding protein (CREB) [100]. In addition to the decrease in the activity of the pro-apoptotic protein Bcl-2, PSs treatment led to an increase in the expression of Bax and procaspase-9. Conversely, another in vivo study reported no changes in the Bax/Bcl-2 ratio after PSs administration [67]. Furthermore, SIT has been reported to exert pro-apoptotic effects in prostate cancer by activating the sphingomyelin cycle [101]. It is well known that PSs inhibit the absorption of cholesterol by competing with it to enter the cell. This process activates Sph synthetase, an enzyme that favors the production of ceramides. Accumulation of ceramides leads to the activation of phosphatase A (PP2A), an enzyme that inhibits Akt (Figure 3).

### 3.3. Phytosterols in BPH—Clinical Evidence

PSs have been studied in a limited number of clinical trials in men with LUTS caused by BPH, but the results are inconclusive. In a randomized, double-blind, placebo-controlled multicenter trial in patients with BPH, patients received SIT (which contained a mixture of PSs) three times/day or a placebo for 6-months. It was shown that SIT was effective, as evidenced by significant improvements in urinary symptoms, urinary flow measures, and QL. There was no significant decline in prostatic volume in either the SIT or placebo groups [102]. In addition to the 6-month trial, an 18-month follow-up study was the only study that examined the long-term effects of PSs on patients with BPH. After 18 months, SIT treatment was continued to provide beneficial effects [103]. Another clinical study reported similar results when using SIT to treat BPH, showing slightly faster changes than those reported by Berges et al. [104]. The studies discussed are summarized in Table 2.

Several plant species were used in the studies as purified extracts, and the dosages ranged from 0.15 mg/day to 130 mg/day. None of the studies specified the use of PSs in a standardized extract form; for a review, see [102,103,104,105,106,107,108]. Using plant extracts containing different SIT dosages may pose problems when combining studies. In these trials, SIT concentrations should be known through the use of standardized extracts. Moreover, the only study that used 100% PSs did not show improvements in men with LUTS attributable to BPH [107]. Another randomized controlled trial demonstrated that *Serenoa repens* extract enriched with 3% PSs has superior efficacy compared to the simple *Serenoa repens* extract in relieving BPH symptoms, thus underlying the importance of PSs [109].

**Table 2:** Clinical studies with PSs effects in HBP.

Type of Study and Subjects	Treatment	Outcomes	Ref.
RCT patients with HBP (n = 200). Duration: 6 months	SIT (20 mg, which contains a mixture of PS), three times/day or placebo	Significant improvement in symptoms score and urinary flow parameters	[102]
RCT patients with HBP (n = 117) Duration: 18 months	SIT (20 mg, which contains a mixture of PS), three times/day or placebo	The effects on QOL of SIT are maintained over at least 18 months in men with symptomatic BPH	[103]
RCT patients with HBP (n = 177) Duration: 6 months	SIT (130 mg) and placebo	SIT is an effective option in the treatment of BPH.	[104]
Single-site study Randomization: noted but method not described (n = 62) Duration: 6 months	SIT (0,15 mg) and placebo	No improvements	[107]
Single-site study Randomization: unclear (n = 80) Duration: 1 month	SIT (65 mg) and placebo	SIT is an effective option in the treatment of BPH	[108]
RCT mild-to-moderate BPH symptoms (n = 99) Duration: 3 months	Saw palmetto oil (3% SIT), Saw palmetto oil (0.2% SIT) and placebo	Efficacy of SIT enriched saw palmetto oil is superior to conventional oil	[109]

The available data from these studies suggest that SIT improves urinary symptoms, flow measures, and QL. All studies were double-blinded and placebo-controlled. The long-term effects of phytosterols on symptomatology or QL have not been extensively studied in studies beyond 18 months. In addition, there is a lack of comparison with conventional therapies (alpha 1-adrenergic receptor antagonists, 5α-reductase inhibitors, or other natural compounds), which are the most widely used and effective drugs for the treatment of BPH. However, to confirm these findings, larger population studies with more robust methodologies are needed.

## 4. Regulatory Framework and Agreement with Label Declaration

Despite extensive research on DS, little attention in public health has focused on challenges in their regulation. There is no evidence of additional benefits with consumption above 3 g/day, and high consumption may have undesirable effects, according to the Scientific Committee on Food of the European Commission; therefore, it is prudent to avoid the consumption of plant sterols of more than 3 g/day [110]. Based on the data reviewed, PSs are generally well tolerated, with few side effects. Several clinical studies have reported adverse reactions to PSs intake, such as flatulence, diarrhea, constipation, nausea, and indigestion [102,111]. Another study demonstrated the safety of long-term consumption of plant sterols (one year) [112].

*Serenoa repens* extract is the main source of PSs used in BPH and is a relatively expensive extract; therefore, it is often falsified [113]. European Union (EU) legislation does not include specific regulations for DS-containing PSs. The United States Pharmacopoeia (USP) requires as an acceptance criterion that the *Serenoa repens* extract contains no less than 0.2% total phytosterols and 0.1% SIT [114]. The same provisions are mentioned in the European Pharmacopoeia (Ph. EUR) [115].

The effective daily dose of PSs for the prevention or treatment of BPH has not been established by any regulatory framework. However, based on a few clinical studies, it is estimated that a dose between 0.060 and 0.140 g of SIT improves symptoms associated with BPH [102,103,104,105,116]. To date, only a few quantitative screening studies of food supplements containing PSs have been conducted. The amounts determined for each study are summarized in Table 3.

The estimated amount of PSs, specifically SIT, in DS-PSs-based treatment for BPH in our reviewed data was in accordance with the estimated amount needed to trigger beneficial effects. Based on the collected data (Table 3), it is evident that the amount of PSs in different DS varied significantly, possibly explained by the lack of a standardized extract of the active principle. In addition, the variability in the content may also be due to the analytical techniques used. The studies listed above used different methods of processing and quantifying samples, except for the study conducted by Sorenson et al. [119]. This was a collaborative study among ten laboratories that analyzed the PSs content of four DS using the same analytical method. Different manufacturers’ PSs contain varying levels of PS but not those produced by the same company [120]. In a study by Cheon Kim et al., the DS content was compared with that of a standardized extract of *Serenoa repens* [118]. PSs levels were 1.4–2 times higher than the reference values. According to the authors of the aforementioned study, the products were adulterated, perhaps by adding less quality extract and sterol-rich exogenous vegetable lipid fractions [118]. Simultaneously, according to the USP and Ph. EUR regulations, not all supplements meet the acceptance criteria [118]. Moreover, in a study conducted by Penugonda et al., DS that exceeded the permissible daily dose of 3 g/day were identified, with a reported maximum PSs of 7.511 g [120]. Other studies have reported the lack or presence of undetectable amounts of plant sterols in some DS with declared PSs content [122].

## 5. Analysis of Phytosterols in Dietary Supplements

### 5.1. Sample Preparation

The first step in characterizing the supplement products is generally represented by the sample preparation. PSs are minor components that typically comprise less than 1% of the matrix (but up to 8% in foods with added PSs or DS). The objectives of this first step are to isolate the sterol fraction and convert all CPs into FPS for final analysis [26].

The extraction method should be selected according to the nature of the matrix, the physical properties of the sample (powder, solution, tincture, etc.), and the form (free or conjugated) in which the plant sterols are found. If we discuss DS, the matrix is a complex one containing both FPS and CPS, but also other organic compounds with similar structures that are extractable by saponification/solvent extraction, such as tocopherols, retinol, and β-carotenes, which can interfere with the quantification of PSs [123]. However, in practice, their levels are low compared to those of PSs, and their effect on the quantification of plant sterols remains statistically uncertain [124].

The DS processing methods reported in the literature are listed in Table 4. The first step involved extracting the lipid fraction from the matrix. Extraction may be carried out using liquid-liquid extraction (LLE) with organic solvents: hexane [125,126], heptan [123], chloroform [127,128], petroleum ether [22,129], or combinations of solvents with different polarities (chloroform-methanol, chlorophorm-methanol-water) [39,130]. This method has the advantages of using simple equipment and low cost of analysis. Solid-phase extraction (SPE) is another commonly used method and is a newer (eco-friendly) technique that provides a more convenient way to extract PSs with minimal analyte loss. Both LLE and SPE can result in recovery yields of 95–100% [131]. Recovery studies should be introduced into the extraction procedures of the analyte of interest to determine the effectiveness of the extraction. The available data also provide other modern extraction procedures suitable for PSs, such as supercritical fluid extraction, microwave-assisted extraction, ultrasound-assisted extraction, ionic liquid extraction, and enzyme-assisted extraction, according to the analyte matrix of interest [132].

**Figure 4:**
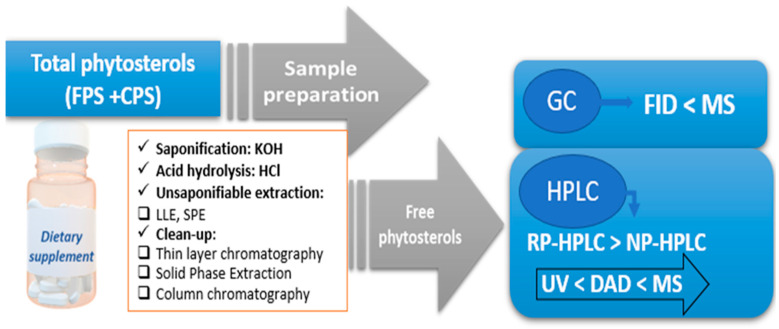
Main steps in the determination of total phytosterols. IS (internal standard) TMS (Trimethylsilyl ether); HPLC (high liquid chromatography); GC (gas chromatography); NP (normal phase); RP (reverse phase).

Owing to the lack of commercial standards for CPS, an intermediate stage involves saponification at room temperature or directly by heating to obtain FPS. Saponification with an ethanol solution of KOH 2M at 80 °C for 30–60 min was noted to be the most commonly used [122]. Sometimes, the glycoside bond in the glycosylated form cannot be hydrolyzed under alkaline conditions, and acid hydrolysis is required [133,134], as shown in Figure 4. The hydrolysis process is followed by the extraction of unsaponifiable lipids by LLE or SPE. Sample processing by alkaline saponification and direct acid hydrolysis without prior extraction of the lipid fraction has also been shown to be an effective method in quantifying the FPS and CPS of *Saccharum officinarum*, using reverse-phase high-performance chromatography (RP-HPLC) as a method of determination [135].

In the final step, the removal of potentially interfering compounds (aliphatic alcohols), isolation, and concentration of the extracts involves methods such as thin-layer chromatography (TLC) or column chromatography (CC). As these analytical methods are time-consuming, they have been replaced by SPE or solid-phase microextraction [131,136]. The main sample preparation sequence is shown in Figure 4.

### 5.2. Determination Methods

A variety of chromatographic methods have been used to characterize and quantify plant sterols isolated from different samples, including gas chromatography (GC) [22,118,119,120,127,128,137,138], thin-layer chromatography (TLC) [23,131,139], and high-performance liquid chromatography (HPLC) [39,122,140,141,142,143]. In addition to classical methods, Fourier transform infrared spectroscopy (FT-NIST), based on previously known reference values obtained by conventional methods, has been introduced for the rapid analysis of total and individual sterols; for a review, see [144,145,146]. Furthermore, Gomez SM et al. reported a physical approach for the quantitative analysis of free sterols and their mixtures in vegetable oils using X-ray powder diffraction and the Rietveld method [147]. In the following sections, the main analytical methods used for monitoring PSs in DS are discussed.

- Gas Chromatography

GC is the standard method for quantifying PSs; for a review, see [23,131,146]. As shown in Table 4, most of the identified studies used GC to analyze DS with PSs content. Instead, for structural identification, GC coupled with mass spectrophotometry (MS) with chemical or electron ionization is an appropriate method for the simultaneous acquisition of both the retention times and molecular masses of the components in the mixture [148]. Furthermore, GC-MS eliminates the problem of co-elution of compounds of interest, which is frequently encountered when determining PSs [22,149].

However, GC presents a laborious sample processing method, including the derivatization of analytes with the formation of trimethylsilyl ethers (TMS) or acetylated derivatives to favor the volatilization of plant sterols [23,146]. The most commonly used derivatizing agents are N-methyl-N-(trimethylsilyl) fluoroacetamide (MTSFA) in anhydrous pyridine and bis-(trimethylsilyl) trifluoroacetamide (BSTFA) with 1% trimethylchlorosilane (TMCS) [23]. The derivatization reaction is difficult to validate because other compounds in the mixture may interact with the derivatization agent. In addition, some degradation products of the derivatization agent may interfere with the chromatographic signal [122]. Furthermore, GC has the disadvantage of requiring high temperatures for analysis, which are not thermally suitable for unstable products, such as plant sterols [122,150,151]. To overcome the effects of fluctuations in instrument operation conditions and other experimental variables, the GC chromatographic peak of a plant sterol is compared to an external or internal standard. Moreover, the correction of the losses of the analyte of interest in quantitative determination during sample processing can be performed using the internal standard [131]. The surrogate internal standards mainly used for quantification of PSs by GC-FID are betuline, 5α-colestanol, and 5β-colestan-3α-ol (epicoprostanol) [23,131].

Due to the structural similarities between PSs and cholesterol, they can be extracted and chromatographically analyzed under similar conditions. Therefore, the Association of Chemical Analysis (AOAC) Official Method 994.10, "Cholesterol in Foods," has been modified and validated for this purpose. The samples were saponified at high temperatures with ethanolic KOH, and the unsaponifiable fractions (SIT, stigmasterol, campesterol) were extracted with toluene. PSs were derivatized to TMS and quantified using GC-FID [121]. Later, an inter-laboratory collaborative study evaluated and recommended the method described above for the determination of PSs from DS at concentrations between 1 and 5 mg/100 g [119].

Currently, the AOAC does not offer an official method for the determination of PSs in different foods, and the previously described determinations have not been validated for the quantification of PSs in fortified/enriched foods and DS with content greater than 1% PSs and do not provide for the identification of plant stanols [119,121]. The methods developed and validated by Laakso et al. and Clement et al. were considered as possible replacement methods, being validated in a single laboratory for a wide range of PSs in a broad range of linearity. Unfortunately, neither method provided adequate GC separation of phytosterols and phytostanols [152,153].

In spite of this, a validated method is available, which uses the traditional sample processing method for the identification of 16 PSs, including the most important ones (SIT, stigmasterol, campesterol) from foods and DS enriched with PSs. This method was adapted for DS as follows: for DS with content greater than 8 g/100 g, it is necessary to use samples smaller than 0.5 g and to increase the amount of solvent for the extraction of the unsaponified fraction (10 mg/mL concentration for total PSs). The application range was between 0.001 g PS/100 g (quantification limit)—55.2 g PS/100 g [22]. The GC methods described in the literature for the determination of sterols from different food matrices or plant products have been published and reviewed by Abdi et al. [131], Lagarda et al. [23], and Garcia-Latas et al. [146].

- High-performance Liquid Chromatography

As an alternative to GC, LC has also been used to determine PSs from various matrices, including DS (Table 4). PSs detection was performed using simple UV [122,142,154] or diode array detection (DAD) [141,155,156,157], corona-charged aerosol detector (CAD) [122,158,158], refractive index (RI) detection [26,146], evaporative light scattering detection (ELSD) [159,160], fluorescence (FL) [161], nuclear magnetic resonance (NMR) [26,146,162], and mass spectrometry (MS) [23,39,141,163]. Reversed-phase HPLC (RP-HPLC) is commonly used for PSs analysis and separation as opposed to normal-phase HPLC (NP-HPLC). For the reversed stationary phase, silica gel supports containing octadecyl (C18)-linked alkyl groups are usually used, whereas the mobile phase is composed of acetonitrile, methanol, or a mixture of water and organic solvents [23,131].

Compared to GC, HPLC methods do not require derivatization, which is an expensive and time-consuming process and can sometimes interfere with the detection method, thus causing the loss of the analyte of interest. However, derivatization is sometimes necessary to optimize the detector response by improving the separation and ionization efficiency in LC-MS [161].

**Table 4:** Determination of total phytosterols in dietary supplements by chromatographic methods.

Method	Detector	Sample Preparation/Extraction	Analytical Methods	Target Compounds	Ref.
GC	FID	Addition internal standard solution Saponification: KOH (2 h/100 °C) Derivatization: BSTFA, and pyridine	Column: (25m × 0.25mm, 0,33 μm, Dimethylpolysiloxane stationary phase) Temperatures (°C): Detector: 325; Injector: 325 Gas (mL/min): Helium (0.5) Injection type: Split ratio, 1:40 Injection volume (μL): 1	campesterol stigmasterol SIT total sterols	[118]
GC	FID/MS	Addition internal standard solution (epicoprostanol) Acid hydrolysis HCl (4M/1 h/100 °C) Lipid extraction: EE: diethyl ether: petroleum ether (50:50/*v*:*v*) Saponification: ethanolic KOH (2 M/80 °C/60 min) Unsaponifiable extraction (LLE): EE (3 times) Derivatization: BSTFA, and pyridine	Column: (30 m × 0.32 mm, 0.25 μm, (poly (94%methyl/5% phenyl) silicone)) Temperatures (°C): column 250, injector 290, detector 290 Gas (mL/min): H2 (1) Injection type Split ratio, 1:25 Injection volume (μL): 1	campesterol stigmasterol SIT	[22]
GC	FID	Alkaline and acid/alkaline protocols Addition internal standard solution (epicoprostanol) Acid hydrolysis: HCl (3N) Saponification: NaOH 2.3 N in MeOH + HCl 3N + NaCl Derivatization: BSTFA, and pyridine	Column: (30 m × 0.32 mm, 0.25 μm) (5%-phenyl)-methylpolysiloxane stationary phase)/(30 m × 0.32 mm, 0.25 μm, (5%-phenyl) (1%-vinyl)-methylpolysiloxane stationary phase) Temperatures (°C): column 250, Injector 290, detector 290 Gas (mL/min): H2 (1) Injection type Split ratio, 1:25 Injection volume (μL): 1	17 phytosterols	[138]
GC	FID/MS	Addition internal standard solution (epicholesterol) Saponification: aqueous KOH (1.3 N/85–89 °C/20 min) Unsaponifiable extraction: cyclohexane Derivatization: (BSTFA/TMCS)	Column: (15 m × 0.32-mm,0.25 μm/5% diphenyl-95%-Dimethylpolysiloxane) Temperatures (°C): column 270, injector 280, detector 300. Gas (ml/min): helium (0.58) Injector type: split ratio, 17:1 Injection volume (μL): 0.5	SIT β-sitosteryl glucoside (BSSG)	[139]
GC	FID	Saponification: ethanolic KOH (1 M/hot plate/80–90 min) Unsaponifiable extraction: toluene Derivatization: TMCS Addition internal standard solution (5 alfa-cholestane)	Column: (25 m × 0.32 mm, 17 μm/5% phenyl-methylsilicone or methyl silicone gum stationary phase) Temperatures (°C): Column 190→255, injector: 250, detector: 300 Gas (mL/min): helium 2/15/3; makeup helium 20/hydrogen 35/air 380 Injection volume (μL): 1	Campesterol Stigmasterol SIT	[119]
GC	FID/MS	Addition internal standard solution (cholestanol), chloroform Saponification: methanol KOH (0.3M/80 °C/60 min) Unsaponifiable extraction: hexane (twice) Derivatization: MTSA, TMCS, and pyridine	Column: (60 m × 0.25 mm, 0.25 um/5% phenyl methyl siloxane stationary phase) Temperatures (°C): column 80→325, detector 230. Gas (mL/min): helium (1) Injection volume (μL): 1	campesterol stigmasterol SIT total sterols	[120]
LC	APCI–MS	Solvent extraction: hexane Saponification with base (ethanolic KOH)/acid hydrolysis Solvent extraction of unsaponifiable material: toluene	1. Column: phenyl (150 mm × 3.9 mm, 3,5 μm) Mobile phase: 58% acetonitrile, 42% water Flow rate (mL/min): 1.1 (isocratic) 2. Column: ACE C18 (150 mm × 3.0 mm, 3 μm) Mobile phase: 90% methanol, 10% water Flow rate (mL/min): 0.80 (isocratic) Injection volume (μL): 5 for qualitative, 10 for quantitative measurement	campesterol, cycloartenol, lupenone, lupeol, SIT, and stigmasterol (Standard Reference Materials containing saw palmetto)	[39]
HPLC	ESI-MS	Saponification: ethanolic KOH (70 °C/30 min) Unsaponifiable extraction: hexane Derivatization: dansyl chloride (40 °C/30 min)	Column: C18 (250 × 3.0 mm, 5 μm) Mobile phase: 95% methanol: 5% water Flow rate (mL/min): 0.5 (isocratic)	campesterol stigmasterol β-sitosterol	[141]
HPLC	UV	Ultrasound-Assisted Emulsification Microextraction (USAEME): Solubilization: methanol Addition: calcium chloride 2mol/L Fat extraction: hexane (heating for 10 min at 50 °C/ultrasonic time 25 min/centrifugation 2600 rpm, 15 min)	Column: RP-18 (125-4 mm, 5 μm) Mobile phase: hexane, propan-1-ol (99.5:0.5; *v*/*v*) Flow rate (mL/min): 0.8−1 (isocratic) Injection volume (μL): 50 UV detection: 212 nm	β-sitosterol	[142]
UHPLC	UV/CAD	Addition internal standard solution (cholesterol) Saponification: ethanolic KOH (80 °C/30 min) Acid hydrolysis: HCl Solvent extraction: hexane (3 min)	Column: (100 × 2.1 mm, 1,7 μm) Phenyl-hexyl Mobile phase: acetonitrile: water Flow rate (mL/min): 0.9 (gradient) Temperature: 60 °C Inj. vol. (μL): 2 Run time (min): 8.5	ergosterol, brassicasterol, campesterol, campestanol, fucosterol, stigmasterol, stigmastanol, SIT esterified form	[122]

GC: gas chromatography; FID: flame ionization detection; BSTFA: bis-(trimethylsilyl) trifluoroacetamide; LLE: Liquid-liquid extraction; MS: mass spectrometry; TMCS: trimethylchlorosilane; MTSFA: N- methyl-N-(trimethylsilyl) fluoroacetamide; LC-liquid chromatography; APCI: atmospheric pressure chemical ionization; ESI: ionization by electrospray; UV: ultraviolet; HPLC: high liquid chromatography; UHPLC: ultra-high liquid chromatography; CAD: Charged aerosol detector.

A further advantage of LC is that it operates at lower temperatures, often at room temperature, making it an ideal method for examining thermally labile compounds, such as PSs; for a review, see [23,122,163], and could be performed by using a non-destructive detector, compared to the FID detector in the GC.

On the other hand, HPLC analysis is associated with many difficulties. If the matrix used is simple and homogeneous (e.g., seed oil), direct HPLC analysis can be applied for the determination of PSs without the laborious step of sample processing. Otherwise, if the analyte of interest is in a complex matrix, preliminary sample purification methods (CC, TLC) are required [131]. Due to the structural similarities, PSs are difficult to separate, requiring increased running time to avoid co-elution. Longer retention times are often associated with the broadening of the chromatographic peaks [122]. Moreover, HPLC-UV or DAD analysis of PSs is associated with sensitivity problems due to the lack of chromophore groups and the broadening of peaks associated with their high lipophilicity at high retention times in RP-HPLC. PSs absorb at wavelengths between 200 and 210 nm. These low wavelengths are not selective; therefore, interference can be observed in the chromatograms by revealing interfering compounds, which may be present in the samples after extraction [145,157].

Recently, a new analytical method, UHPLC with tandem DAD (UV)/charged aerosol detector (CAD), allows rapid determination (<8.5 min) and efficient chromatographic separation of PSs from DS. It has been demonstrated that CAD sensitivity is three times greater than that of UV at wavelengths below 210 nm [122].

However, LC-MS can address many of the challenges encountered in previous analytical strategies due to the selectivity and specificity of the detection method [23,163]. The most common types of MS detectors for PSs analysis based on the ionization source are electrospray ionization (ESI), atmospheric pressure chemical ionization (APCI), atmospheric pressure photoionization (APPI), matrix-assisted laser desorption ionization (MALDI), and ambient MS, such as direct analysis in real time (DART) [163]. Because PSs are extremely lipophilic with few polar functional groups, ESI, which is one of the most widespread and powerful ionization techniques, is unsuitable for their determination [23,141,164,165]. Thus, other ionization techniques, such as APCI or APPI [39,141,166], were applied. On the other hand, quantitative MS-based analysis from complex matrices is associated with an unpredictable matrix effect and increased cost of the internal standard [167].

A recent paper reported a fast, simple, and low-cost dansylation derivatization method that solves previously encountered problems. The optimal derivatization reaction conditions consisted of dichloromethane as the solvent and 4-dimethylaminopyridine as the catalyst at 40 °C for 30 min. The derivatization process by dansylation allows the improvement of UV detection (254 nm), the limitation of the detection of non-target compounds, and the separation by RP-HPLC, with the exception of stigmasterol and campesterol. Moreover, this method solves the problem of ESI inefficiency, which leads to increased reproducibility and linearity [141].

## 6. Conclusions

This review summarizes the potential effects of PSs intake on BPH by presenting the thus far published data obtained from in vitro studies, animal studies, and clinical trials. BPH prevention and treatment require a detailed understanding of the molecular mechanisms underlying PSs-induced activities. The use of PSs for BPH is primarily explained by their anti-androgenic activity through 5-αR inhibition but can also act via apoptotic pathways linked to the endocrine system. Recently, the anti-inflammatory and antioxidant actions of PSs have emphasized their potential for use as individual active principles in BPH. Considering that the quantity and quality of PSs in DS are intrinsically related to their efficacy, the data on monitoring PSs content in DS has been reviewed. Substantial variability in botanical supplement composition and concentrations has been noted. In the future, the use of standardized, rapid, and economic analysis techniques may allow the implementation of a system to certify the authenticity of PSs content in DS used in therapy. Overgeneralization of pharmacological effects of all plant-mixture without any specific emphasis on PSs compounds and the shortage of any valid regulatory framework is also considered a significant challenge.

To conclude, PSs should be considered with other medical therapies for patients with symptomatic BPH, but lack of standardization and pharmacological effect limits the potential clinical usefulness. Thus, further studies are needed to ensure a more in-depth understanding of the molecular mechanisms underlying PSs-induced activities and to design upcoming strategies to overcome the currently identified regulation and analytical-related gaps.

## Figures and Tables

**Table 1:** Dietary components with 5-αR inhibitory effect.

Molecules	Sample	IC_50_ μg/mL	Ref.
Sitosterol Finasteride	standardized extract standard	1.1 0.003	[59]
Stigmasterol	*Phyllanthus urinaria*, extract from the whole plant	11.2	[60]
**Plant extract with PSs content**			
*Serenoa repens*	whole plant	25–2200	[65]
*Epilobium parviflorum*	stem and leaves	160	[61]
*Pygeum africanum*	standardized extract	780	[62]
*Curcubita pepo*	pupmkin seed oil and soft extract	5880	[63]
*Urtica dioica*	standardized extract	14,700	[62]

**Table 3:** Comparison of levels of phytosterols in dietary supplements between different studies.

Phytosterols Percentage of Sterol in Total Extract % (*w*/*w*)				
β-Sitosterol	Stigmasterol	Campesterol	Total Sterols	References
0.030–0.200	0.004–0.044	0.009–0.062	0.055–0.308	[117]
0.249–0.271	0.081–0.166	0.057–0.144	0.420–0.585	[118]
0.141–0.388	0.011–0.136	0.038–0.155	NR	[119]
0.010–3.255	0.001–1.975	0.003–2.228	0.015–7.511	[120]
0.001–0.276	0.008–0.104	0.022–0.117	NR	[121]
0.1	NR	NR	0.2	EU/USP monograph [114,115]
0.041	0.004	0.010	NR	SRM 3250 [39]
0.164	0.022	0.050	NR	SRM 3251 [39]

NR—not reported.

## Data Availability

Not applicable.
